# The Pareidolia Test: A Simple Neuropsychological Test Measuring Visual Hallucination-Like Illusions

**DOI:** 10.1371/journal.pone.0154713

**Published:** 2016-05-12

**Authors:** Yasuyuki Mamiya, Yoshiyuki Nishio, Hiroyuki Watanabe, Kayoko Yokoi, Makoto Uchiyama, Toru Baba, Osamu Iizuka, Shigenori Kanno, Naoto Kamimura, Hiroaki Kazui, Mamoru Hashimoto, Manabu Ikeda, Chieko Takeshita, Tatsuo Shimomura, Etsuro Mori

**Affiliations:** 1 Department of Behavioral Neurology and Cognitive Neuroscience, Tohoku University School of Medicine, Sendai, Japan; 2 Department of Occupational Therapy, Yamagata Prefectural University of Health Sciences, Yamagata, Japan; 3 Department of Speech, Language and Hearing Sciences, Niigata University of Health and Welfare, Niigata, Japan; 4 Department of Neurology, South Miyagi Medical Center, Ohgawara, Japan; 5 Department of Neuropsychiatry, Kochi Medical School, Nangoku, Japan; 6 Department of Psychiatry, Osaka University School of Medicine, Suita, Japan; 7 Department of Neuropsychiatry, Faculty of Life Sciences, Kumamoto University, Kumamoto, Japan; 8 Department of Rehabilitation Medicine, Akita Prefectural Center of Rehabilitation and Psychiatric Medicine, Daisen, Japan; University of Antwerp, BELGIUM

## Abstract

**Background:**

Visual hallucinations are a core clinical feature of dementia with Lewy bodies (DLB), and this symptom is important in the differential diagnosis and prediction of treatment response. The pareidolia test is a tool that evokes visual hallucination-like illusions, and these illusions may be a surrogate marker of visual hallucinations in DLB. We created a simplified version of the pareidolia test and examined its validity and reliability to establish the clinical utility of this test.

**Methods:**

The pareidolia test was administered to 52 patients with DLB, 52 patients with Alzheimer’s disease (AD) and 20 healthy controls (HCs). We assessed the test-retest/inter-rater reliability using the intra-class correlation coefficient (ICC) and the concurrent validity using the Neuropsychiatric Inventory (NPI) hallucinations score as a reference. A receiver operating characteristic (ROC) analysis was used to evaluate the sensitivity and specificity of the pareidolia test to differentiate DLB from AD and HCs.

**Results:**

The pareidolia test required approximately 15 minutes to administer, exhibited good test-retest/inter-rater reliability (ICC of 0.82), and moderately correlated with the NPI hallucinations score (r_s_ = 0.42). Using an optimal cut-off score set according to the ROC analysis, and the pareidolia test differentiated DLB from AD with a sensitivity of 81% and a specificity of 92%.

**Conclusions:**

Our study suggests that the simplified version of the pareidolia test is a valid and reliable surrogate marker of visual hallucinations in DLB.

## Introduction

Dementia with Lewy bodies (DLB) is the second most common form of neurodegenerative dementia, and it accounts for ~9.7% of people with dementia in population-based studies and ~24.7% of people with dementia in clinic-based studies [1]. Patients with DLB suffer more severe functional impairments and a higher mortality rate than patients with Alzheimer's disease (AD) [2,3]. Caregiver burden and distress are also greater in DLB than AD, which leads to earlier institutionalization of patients with DLB in nursing homes [2,4]. Therefore, patients with DLB are associated with higher healthcare costs than those with AD [5]. Early diagnosis and intervention are required to improve the quality of life of patients and caregivers and to reduce the economic burden to society, but this need is currently unmet. Previous clinico-pathological studies demonstrated that the specificity of the clinical diagnostic criteria for DLB is consistently high (over 90%), but its sensitivity is extremely low (12.1–83.0%) [6–8], suggesting that underdiagnosis is a significant issue.

Visual hallucinations are one of the three core symptoms in the diagnostic criteria for DLB [9,10], and are observed in over 70% of clinically diagnosed DLB patients [11,12]. This symptom correlates with cortical Lewy body burden and cholinergic degeneration in the neocortex [13,14]. Therefore, the measurement of visual hallucinations is important in the differential diagnosis and prediction of treatment response. Currently, the identification and quantification of visual hallucinations relies on self or proxy reports. Structured interview methods, such as the Neuropsychiatric Inventory (NPI) and North-East Visual Hallucinations Interview, are widely used [15,16]. These methods are helpful and well validated, but they are difficult to use in patients who have no reliable informants or who lack insight into their own symptoms. Methods to detect and measure visual hallucinations in more direct ways would be helpful.

Visual hallucinations are defined as false perception that arise independently from actual visual scenes, whereas visual illusions are misperceptions that result from distortion of actual visual scenes. However, the distinction between visual hallucinations and visual illusions are often ambiguous because patients see things whenever they are awake and have their eyes open. Pareidolias are visual illusions of meaningful objects which arise from ambiguous forms embedded in visual scenes. We recently developed a tool that evokes and measures pareidolias, which is called the pareidolia test, and demonstrated phenomenological similarities between visual hallucinations and pareidolias in DLB [17,18]. In addition, the numbers of illusory responses in the pareidolia test were significantly correlated with the severity of visual hallucinations that was measured by the NPI, suggesting the usefulness of the pareidolia test as a surrogate indicator of visual hallucinations [17,18].

We have previously developed two different versions of the pareidolia test, the scene and noise pareidolia tests. The scene pareidolia test exhibits high sensitivity to discriminate DLB from AD, but it does not correlate with clinical visual hallucinations [17]. The noise pareidolia test correlates well with visual hallucinations, but it does not exhibit sufficient sensitivity [18]. The distinct noso- and psychometric profiles of the scene and noise pareidolia tests suggest that they may reflect different aspects of psychological or neurological mechanisms of pareidolias and may be complementary to each other. Therefore, the combined use of both versions may improve the clinical utility of the test. In the current study, we created a simplified, clinically usable version of the pareidolia test based both on the scene and noise versions of the test and examined its validity and reliability.

## Methods

### Participants

We recruited 52 patients with probable DLB (age, 79.5±7.2 years; 31 females; disease duration, 2.7±2.0 years; median (range) of the Clinical Dementia Rating (CDR), 1 (0.5–2); the Mini-mental State Examination (MMSE) score, 19.8±4.8) and 52 patients with probable AD (age, 79.8±6.2 years; 39 females; disease duration, 3.0±1.6 years; median (range) of the CDR, 1 (0.5–2); the MMSE score 19.6±3.8) from dementia specialty clinics (**Table 1**). Twenty control subjects (age, 78.8±5.0 years; 15 females; the MMSE score 28.0±1.1) were recruited from the local community through an advertisement. None of the subjects had participated in our previous studies [17,18]. The three groups were comparable in age, sex, education and visual acuity. The severity of cognitive impairment was assessed using the MMSE [19], and it was matched between the DLB and AD groups. All patients underwent an examination by experienced behavioral neurologists or psychiatrists, an MRI and routine laboratory investigations. DLB was diagnosed according to the international workshop criteria of DLB [9], and AD was diagnosed based on the standard guidelines set by the National Institute of Neurological and Communicative Disorders and Stroke and the Alzheimer's Disease and Related Disorders Association [20]. The following exclusion criteria were used: (1) a history of other neurological, psychiatric or severe ocular diseases, (2) a best-corrected acuity of less than 20/70 and (3) language deficits that hinder task execution. At the time of examination, 19 patients with DLB and 21 patients with AD were treated with a cholinesterase inhibitor (Chi-square test, p = 0.32). Three patients with DLB and none patients with AD were treated with a levodopa (Chi-square test, p = 0.08). None of the patients took antidepressants or antipsychotics. In the DLB group, 29 patients had cognitive fluctuations, 39 patients had recurrent visual hallucination and 40 patients had parkinsonism.

**Table 1:** Demographic and clinical profiles of the participants.

		DLB (n = 52)	AD (n = 52)	HC (n = 20)	p-value
Sex (female/male) ^a^		31/21	39/13	15/5	0.19
Age, years		79.5 (7.2)	79.8 (6.2)	78.8 (5.0)	0.83
Education, years		10.5 (2.4)	10.1 (2.6)	11.4 (2.1)	0.13
Disease duration, years ^b^		2.7 (2.0)	3.0 (1.6)		0.54
Visual acuity ^c^		20/32 (20/40-20/32)	20/32 (20/40-20/25)	20/32 (20/40-20/25)	0.09
CDR score ^d^		1 (0.5–2)	1 (0.5–2)		0.07
***Neuropsychology***					
MMSE		19.8 (4.8)	19.6 (3.8)	28.0 (1.1)	**< 0.01** ^**f**^^,^ ^**g**^
ACE-R					
	Total	59.6 (15.1)	61.4 (13.4)	93.3 (4.0)	**< 0.01** ^**f**^^,^ ^**g**^
	Attention/Orientation	12.3 (3.2)	12.4 (3.0)	17.4 (0.6)	**< 0.01** ^**f**^^,^ ^**g**^
	Memory	10.6 (5.1)	7.9 (3.5)	22.6 (3.3)	**< 0.01** ^**e**^^,^ ^**f**^^,^ ^**g**^
	Verbal fluency	6.2 (3.7)	7.7 (3.6)	12.9 (1.2)	**< 0.01** ^**f**^^,^ ^**g**^
	Language	20.5 (3.9)	20.5 (4.0)	24.9 (1.3)	**< 0.01** ^**f**^^,^ ^**g**^
	Visuospatial	10.0 (3.9)	12.9 (3.5)	15.6 (0.8)	**< 0.01** ^**e**^^,^ ^**f**^^,^ ^**g**^
Shape detection		17.7 (1.9)	18.7 (1.3)	19.6 (0.6)	**< 0.01** ^**e**^^,^ ^**f**^
Position discrimination		17.5 (2.9)	18.7 (1.6)	19.5 (0.6)	**< 0.01** ^**e**^^,^ ^**f**^
Face recognition		23.8 (3.6)	26.2 (3.2)	28.1 (1.4)	**< 0.01** ^**e**^^,^ ^**f**^
***Neuropsychiatric inventory*** ^**d**^** **					
Persecutory delusions		0 (0–2)	0 (0–0)		0.07
Delusional misidentifications		0 (0–3)	0 (0–0)		**< 0.01**
Hallucinations		3 (0–8)	0 (0–0)		**< 0.01**
Agitation/aggression		0 (0–1)	0 (0–2)		0.26
Dysphoria		0 (0–1)	0 (0–2)		0.76
Anxiety		0 (0–3)	0 (0–0)		0.20
Euphoria		0 (0–0)	0 (0–0)		0.31
Apathy		3 (0–4)	3 (0–4)		0.66
Disinhibition		0 (0–0)	0 (0–0)		0.42
Irritability/lability		0 (0–0)	0 (0–1)		0.97
Aberrant motor behavior		0 (0–3)	0 (0–1)		0.10
Fluctuations in cognition		3 (0–8)	0 (0–0)		**< 0.01**

The visual acuity and NPI scores are indicated as medians (interquartile range). The CDR score is indicated as median (range). The other values indicate the mean (standard deviation). Significant p-values are indicated in bold.

DLB, Dementia with Lewy Bodies; AD, Alzheimer’s disease; HC, healthy controls.

^a^ Chi-square test

^b^ Student’s-t test

^c^ Kruskal–Wallis test

^d^ Mann–Whitney U test

^e^ DLB < AD, or DLB > AD (Scheffé’s test, p < 0.05)

^f^ DLB < HC (Scheffé’s test, p < 0.05)

^g^ AD < HC (Scheffé’s test, p < 0.05); One-way analysis of variance was used for other variables.

The ethical committee of the Tohoku University Hospital approved all procedures in this study. All participants provided written informed consent after receiving a detailed explanation of the study.

### Background Neuropsychological and Behavioral Assessments

The Addenbrooke's Cognitive Examination-Revised (ACE-R) provides the domain scores for attention/orientation, memory, verbal fluency, language, and visuospatial function, and this test was used to assess various cognitive domains [21,22]. We also used the Shape Detection Screening and Position Discrimination subtests of the Visual Object and Space Perception battery [23] and the Face Recognition subtests (face-to-face matching of unknown faces, same/different judgment of unknown faces in different views, gender and age judgments of unknown faces) of the Visual Perception Test for Agnosia to assess visuoperceptual and visuospatial functions [24]. The MMSE and ACE-R total scores were used as measures of global cognitive function. The Clinical Dementia Rating (CDR) was used to assess the global severity of cognitive impairment [25].

The NPI was administered to patient caregivers [15]. The original NPI consist of the following 10 behavioral domains: delusions, hallucinations, dysphoria, anxiety, agitation/aggression, euphoria, disinhibition, irritability/lability, apathy, and aberrant motor behavior. We made several modifications to the original NPI. First, the ‘delusion’ domain was separated into two different categories: persecutory delusions and delusional misidentifications. Second, we employed an additional domain for fluctuations in cognition (cognitive fluctuation inventory) [26]. Twelve domains of neuropsychiatric symptoms were evaluated based on the clinical status of patients during the past month. The frequency (range: 1–4), severity (1–3) and domain total scores (the product of the frequency score multiplied by the severity score) were recorded for each behavior.

### The Pareidolia Test

We previously devised two versions of the tests, the scene and noise pareidolia tests, to evoke and measure pareidolic illusions. The scene pareidolia test originally consisted of 25 blurred natural scene images. We created an abbreviated version in the current study that consisted of the 10 images that produced illusory responses most frequently in our previous study [17]. A detailed explanation and two training trials were given immediately before administering the test. Subjects were instructed to point to and describe in as much detail as possible the objects shown on each image. Each image was presented for a maximum of 60 seconds. No feedback was given to subjects, regardless of whether the responses were correct. Subjects’ responses were classified into three types: (1) correct responses; (2) illusory responses, in which subjects falsely identified objects that were not on the images; and (3) other responses, in which subjects provided no response or said “I don’t know”. The correct answers for each image were defined a priori. When subjects responded with such comments as “It looks like X”, we asked the subjects whether the object (X) was actually in the picture or whether the subject saw something that just looked similar to X. Only the former type of responses was regarded as illusory responses. The number of all illusory responses was used as a measure of pareidolic illusions in our previous study. However, we counted the number of images on which subjects made one or more illusory responses in the current study to ease test administration and scoring (maximum score of 10).

The noise version contained 40 black and white images that consist of visual noise with a spatial frequency of 1/f^3^. Black and white images of human faces were embedded in 8 of the 40 images. Subjects were requested to state whether a face was present and point to the place where they observed a face after a detailed explanation and three training trials were given. Each picture was presented for a maximum of 30 seconds. No feedback was given to subjects, regardless of whether the responses were correct. The responses were classified into three types: (1) illusory responses, in which subjects falsely found faces in images without a face; (2) detection misses, in which subjects did not detect the embedded faces, and (3) correct responses, in which subjects correctly responded “nothing exists” to the noise stimuli or correctly detected the embedded image in the images that contained faces. The number of images in which subjects made illusory responses was used as a measure of pareidolic illusions [18]. In scoring pareidolic responses, we originally used images with a face and images without a face [17]. In the current study, we used only the 32 images without a face to ease scoring (maximum score of 32).

We defined the pareidolia score as the sum number of images with illusory responses in the scene and noise versions.

### Reliability and Validity

Fifteen patients with DLB and 15 patients with AD were given the pareidolia test twice in an interval of 27.0 ± 13.1 days to assess the test-retest reliability. Two different examiners administered the first and second sessions. Three examiners were involved in test administration, and the order of examiners was counterbalanced across subjects.

The NPI hallucinations score was used as a reference test in 52 patients with DLB to investigate the validity of the pareidolia test as a surrogate indicator of visual hallucinations.

### Statistical Analysis

Intergroup comparisons of the pareidolia test and other neuropsychological tests were performed using one-way analysis of variance (ANOVA) with post-hoc Scheffé’s test or the Kruskal-Wallis test and post-hoc Mann-Whitney U test with Bonferroni correction where appropriate. The Mann-Whitney U test was used to compare NPI subscores between the DLB and AD groups. The test-retest and inter-rater reliability and the concurrent validity of the pareidolia test were assessed using the intra-class correlation coefficient (ICC) and Spearman’s rank correlation, respectively. A receiver operating characteristic (ROC) analysis was used to evaluate the sensitivity and specificity of the pareidolia test to differentiate DLB from AD or controls, and the optimal cut-off score was determined to maximize the sum of sensitivity and specificity. We calculated the area under the curve (AUC) to compare the pareidolia test with other neuropsychological tests on the ability to discriminate between DLB and AD. The relationship between the performance on the pareidolia test and other neuropsychological/behavioral variables was assessed using Pearson's correlation coefficient or Spearman's rank correlation.

## Results

### Background Neuropsychological and Behavioral Assessments

The results are summarized in **Table 1**. The DLB group was worse than the AD group on the ACE-R visuospatial subscore, shape detection screening test, position discrimination test and face recognition test, but the DLB group achieved better ACE-R memory scores than the AD group. No significant differences were observed between the DLB and AD groups on the other neuropsychological tests. The DLB group exhibited significantly higher NPI subscores for delusional misidentifications, hallucinations and fluctuations in cognition than the AD group.

### Performances on the Pareidolia Test in Dementia with Lewy Bodies, Alzheimer's Disease and Healthy Controls

**Fig 1:**
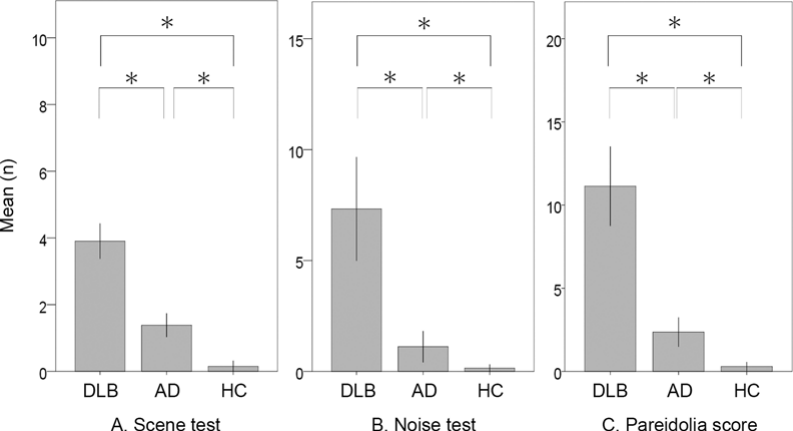
(A) Numbers of images in which subjects made illusory responses in the scene pareidolia test. (B) Numbers of illusory responses in the noise pareidolia test. (C) The pareidolia score. Significance is denoted by an asterisk (Mann-Whitney U test, p < 0.05/3).

The mean (standard deviation, SD) numbers of images in which subjects made illusory responses in the scene pareidolia test were 3.9 (1.9) for DLB, 1.4 (1.3) for AD and 0.2 (0.4) for HC (**Fig 1A**). The DLB group produced more illusory responses than the AD (p < 0.01) and HC groups (p < 0.01), and the AD group produced more illusory responses than the HC group (p < 0.01). The numbers of images in which correct responses were made were 7.0 (2.0) for the DLB group, 7.9 (1.6) for the AD group and 9.5 (0.8) for the HC group. The AD group produced more correct responses than the DLB (p = 0.03), and the HC group produced more correct responses than the DLB (p < 0.01) and AD groups (p < 0.01). The mean numbers of illusory responses in the scene pareidolia test were 4.1 (2.0) for DLB patients with hallucinations and 3.3 (1.3) for those without hallucinations (p = 0.24) (**S1 Fig**).

The mean numbers of illusory responses in the noise pareidolia test were 7.3 (8.4) for DLB, 1.1 (2.5) for AD and 0.2 (0.4) for HC (**Fig 1B**). The DLB group produced more illusory responses than the AD group (p < 0.01) and HC group (p < 0.01), and the AD group produced more illusory responses than the HC group (p = 0.03). The numbers of correct responses were 31.8 (8.8) for DLB, 38.5 (2.5) for AD and 39.9 (0.4) for HC. Significant differences were observed in all pair-wise comparisons between groups (p < 0.01). The numbers of detection misses were 1.2 (1.5) for DLB, 0.5 (0.8) for AD and 0.0 (0.0) for HC. Significant differences were observed between the DLB and AD groups (p = 0.01), the DLB and HC groups (p < 0.01), and the AD and HC groups (p < 0.01). The mean numbers of illusory responses in the noise pareidolia test were 8.6 (8.9) for DLB patients with hallucinations and 2.4 (3.1) for those without hallucinations (p = 0.01) (**S1 Fig**).

The mean pareidolia scores were 11.1 (8.6) for DLB, 2.4 (3.2) for AD and 0.3 (0.6) for HC (**Fig 1C**). Significant differences were observed between the DLB and AD groups (p < 0.01), the DLB and HC groups (p < 0.01) and the AD and HC groups (p < 0.01). The mean pareidolia scores were 12.6 (9.0) for DLB patients with hallucinations and 5.7 (3.1) for those without hallucinations (p = 0.01) (**S1 Fig**).

### Reliability and Validity of the Pareidolia Test

The results are summarized in **Table 2**. The time required to administer the scene pareidolia test was 9.8 ± 1.5 minutes for the dementia patients and 9.6 ± 0.7 minutes for the HCs. The noise pareidolia test required 5.5 ± 3.0 minutes for the dementia patients and 2.3 ± 0.2 minutes for the HCs.

**Table 2:** Characteristics of the scene pareidolia test, noise pareidolia test and pareidolia score.

	Scene test	Noise test	Pareidolia score
Administration time; min (SD)	9.8 (1.5)	5.5 (3.0)	15.4 (3.9)
Test-retest/inter-rater reliability^a^	0.50	0.82	0.82
Correlation with the NPI hallucinations score (r_s_) ^b^	0.17	0.41	0.42
Differentiation between DLB and AD; Sensitivity/Specificity (Cut-off score) ^c^	0.92/0.58 (1/2)	0.60/0.92 (2/3)	0.81/0.92 (4/5)

^a^ Intra-class correlation coefficient

^b^ Spearman's rank correlation

^c^ Receiver operating curve characteristic analysis.

The ICC for illusory responses in the scene pareidolia test was 0.50 (F = 3.0, p < 0.01), which indicates a fair test-retest/inter-rater reliability, but the ICCs for illusory responses on the noise pareidolia test and pareidolia score were 0.82 (F = 10.1, p < 0.01) and 0.82 (F = 10.4, p < 0.01), respectively, which indicates excellent reliabilities.

There was no significant correlation between illusory responses in the scene pareidolia test and NPI hallucinations score in the DLB patients (r_s_ = 0.17, p = 0.22), but illusory responses in the noise pareidolia test (r_s_ = 0.41, p < 0.01) and pareidolia score (r_s_ = 0.42, p < 0.01) were significantly correlated with the NPI hallucination score.

In our previous study, we counted the total number of illusory responses for a primary measure in the scene pareidolia test [17]. For a comparison, we applied the same analysis to the current data. As a result, the current and previous scoring procedures of the scene pareidolia test were equivalent in test-retest/inter-rater reliability, correlation with the NPI hallucinations score and discrimination ability between DLB and AD (**S1 Table**).

### Differentiation between Dementia with Lewy Bodies and Alzheimer's Disease

**Fig 2:**
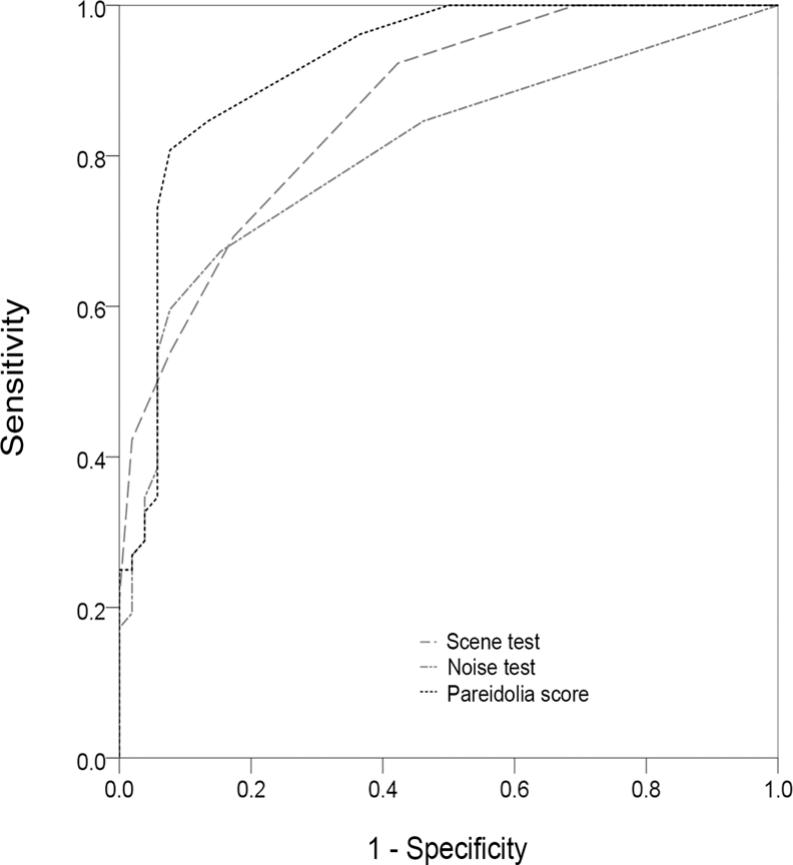
Receiver Operating Characteristic analysis for the pareidolia test to differentiate DLB from AD. The areas under the curve (AUCs) were 0.86 for the scene pareidolia test, 0.82 for the noise pareidolia test and 0.92 for the pareidolia score.

The results are summarized in **Table 2** and **Fig 2**. ROC analyses demonstrated that the optimal cut-off scores for the scene and noise pareidolia tests were 1/2 and 2/3, respectively. The sensitivity/specificity of the scene and noise pareidolia tests were 92%/58% and 60%/92%, respectively, using these cut-off scores. The optimal cut-off score for the pareidolia score was 4/5, and the sensitivity/specificity was 81%/92%.

The AUCs for the scene pareidolia test, noise pareidolia test and pareidolia score were 0.86 (95% CI: 0.79–0.93), 0.82 (0.74–0.90) and 0.92 (0.86–0.97), respectively. The AUCs of the neuropsychological and behavioral measures in which significant differences were observed between the DLB and AD groups were as follows: 0.52 (0.40–0.63) for the MMSE score, 0.54 (0.43–0.65) for the ACE-R total score, 0.66 (0.55–0.76) for the ACE-R memory score, 0.72 (0.63–0.82) for the ACE-R visuospatial score and 0.69 (0.59–0.79) for the sum score of the visuoperceptual and visuospatial tests. The AUCs for NPI subscores of delusional misidentifications, hallucinations, and fluctuations in cognition were 0.68 (0.58–0.78), 0.86 (0.78–0.94), and 0.78 (0.69–0.87), respectively.

**Table 3** and **S2 Table** show the numbers of patients who scored above or below the cut-off scores on the scene and noise pareidolia tests and pareidolia score.

**Table 3:** Numbers of patients who scored above or below the cut-off scores on the noise and scene pareidolia tests.

		DLB			AD	
	Scene test (+)	Scene test (-)	Total	Scene test (+)	Scene test (-)	Total
Noise test (+)	31	4	35	4	0	4
Noise test (-)	17	0	17	18	30	48
Total	48	4	52	22	30	52

The cut-off scores of the scene and noise pareidolia tests were 1/2 and 2/3, respectively.

### Differentiation between Dementia with Lewy Bodies and Healthy Controls

The optimal cut-off scores for the scene pareidolia, noise pareidolia and pareidolia score in the discrimination between DLB and HCs were 1/2, 0/1 and 2/3, respectively. The sensitivity/specificity of the scene pareidolia, noise pareidolia and pareidolia score using these cut-off scores were 92%/100%, 85%/85% and 96%/100%, respectively.

### Correlation between the Pareidolia Test and Other Clinical Variables in Patients with Dementia with Lewy Bodies

The results are summarized in **Table 4**. The scene pareidolia test, noise pareidolia test and pareidolia score were not significantly correlated with age, education or visual acuity. A significant correlation was observed between the scene pareidolia test and ACE-R visuospatial scores, but neither the noise pareidolia nor pareidolia score was significantly correlated with any neuropsychological measures. There was no significant correlation between the scene pareidolia and NPI scores. The noise pareidolia test and pareidolia score exhibited the strongest correlations with the NPI hallucinations score compared with other neuropsychological and behavioral measures.

**Table 4:** Correlation between the pareidolia test and other clinical variables in DLB patients.

		Scene test		Noise test		Pareidolia score	
		r, r_s_	p-value	r, r_s_	p-value	r, r_s_	p-value
Age ^a^		0.06	0.65	-0.22	0.12	-0.20	0.17
Education ^a^		-0.11	0.43	-0.01	0.94	-0.03	0.84
Disease duration ^a^		0.01	0.93	-0.01	0.92	-0.01	0.93
Visual acuity ^b^		-0.17	0.23	0.06	0.68	-0.05	0.73
CDR score ^b^		0.25	0.07	0.09	0.54	0.14	0.32
***Neuropsychology*** ^a^							
MMSE		-0.10	0.48	-0.18	0.21	-0.20	0.15
ACE-R							
	Total	-0.17	0.24	0.04	0.80	-0.01	0.99
	Attention/Orientation	-0.09	0.55	-0.17	0.24	-0.17	0.22
	Memory	-0.03	0.83	-0.04	0.79	-0.04	0.77
	Verbal fluency	-0.03	0.99	0.24	0.09	0.24	0.09
	Language	-0.23	0.10	0.17	0.22	0.11	0.43
	Visuospatial	**-0.30**	**0.03**	-0.08	0.59	-0.15	0.29
Shape detection		-0.27	0.05	-0.15	0.30	-0.21	0.13
Position discrimination		-0.16	0.26	0.07	0.63	0.04	0.77
Face recognition		-0.23	0.10	-0.01	0.99	-0.07	0.61
***Neuropsychiatric inventory*** ^**b**^							
Persecutory delusions		0.20	0.89	0.20	0.15	0.15	0.31
Delusional misidentifications		0.26	0.06	0.12	0.39	0.17	0.24
Hallucinations		0.17	0.22	**0.41**	**<0.01**	**0.42**	**<0.01**
Agitation/aggression		0.26	0.07	**0.30**	**0.03**	0.27	0.06
Dysphoria		-0.07	0.75	0.12	0.39	0.10	0.50
Anxiety		-0.05	0.51	0.26	0.06	0.21	0.13
Euphoria		0.03	0.83	-0.03	0.98	-0.01	0.92
Apathy		0.05	0.74	0.18	0.21	0.19	0.18
Disinhibition		-0.04	0.78	0.06	0.67	-0.02	0.89
Irritability/lability		-0.05	0.97	**0.29**	**0.03**	0.24	0.09
Aberrant motor behavior		0.15	0.31	0.19	0.17	0.20	0.15
Fluctuations in cognition		-0.27	0.06	0.26	0.06	0.18	0.21

^a^ Pearson’s correlation coefficient

^b^ Spearman’s rank correlation coefficient.

Significant p-values are indicated in bold.

## Discussion

The two versions of the pareidolia test, the scene and noise pareidolia tests, were developed previously [17,18]. Although both test versions evoked phenomenologically similar pareidolic illusions, most of which were images of humans and animals, they exhibited distinct noso- and psychometric profiles. The scene pareidolia test demonstrated an excellent ability to discriminate DLB from AD, but it was poorly correlated with clinical visual hallucinations [17]. The noise pareidolia test correlated well with visual hallucinations, but its utility for differentiating between DLB and AD was inferior to the scene pareidolia test [18]. The scene and noise pareidolia tests may reflect different aspects of psychological or neurological mechanisms of pareidolias. We combined both tests in the current study to take advantage of the assets of each test version. We also abbreviated the test and simplified its administration and scoring procedures to improve its usefulness in clinical settings. The pareidolia score, a composite score of the scene and noise pareidolia tests, exhibited an excellent inter-rater/test-retest reliability, good correlation with clinical hallucinations and a better balance of sensitivity and specificity compared with each of the two test versions alone (**Table 2**). The mean administration time was approximately 15 minutes, which is acceptable in clinical settings. Therefore, the current version of the pareidolia test may be a helpful tool in dementia clinics.

The pareidolia score and noise pareidolia test significantly correlated with the NPI hallucinations score but were not correlated with measures of global cognitive ability, such as the MMSE and ACE-R total scores. The pareidolia score and noise pareidolia test may serve as a reliable surrogate marker of visual hallucinations in dementia patients with mild to moderate cognitive impairment, which was the level of cognitive severity of subjects in the current study. The pareidolia score exhibited better sensitivity to discriminate DLB from AD compared with the noise pareidolia test alone. However, the use of the noise pareidolia test independently may be a good option to measure treatment responses because the noise pareidolia test exhibited a good correlation with clinical visual hallucinations. By contrast, we found no significant correlation between the scene pareidolia test and NPI hallucinations score, which is consistent with a previous study [17]. This result likely occurred because not only DLB patients with frank hallucinations but also those without visual hallucinations produced illusory responses in this test. The reduced number of test stimuli and simplification of the scoring procedure may also have reduced the quantitativeness of the test at the expense of the ease of test administration. A subset of AD patients produced as many illusory responses as DLB patients (**Table 3**). The low sensitivity of the current clinical diagnostic criteria for DLB suggest that this result does not merely represent false positives, but it may predict the future development of full-blown DLB symptoms [8,9,27]. The prediction ability of the scene pareidolia test for conversion to clinical DLB should be addressed in future studies.

The inter-rater/test-retest reliabilities of the pareidolia score and noise pareidolia test were excellent, but the reliabilities of the scene pareidolia test remained moderate. We conducted two supplementary analyses to identify the factors that were associated with inter-rater/test-retest variability. In the first analysis, ICCs for the scene pareidolia test, noise pareidolia test and pareidolia score were calculated separately in 15 DLB patients and 15 AD patients. In the second analysis, we presented the identical video-recorded patient responses (5 DLB and 5 AD) in the scene pareidolia test to two examiners and compared their scores. ICC was significantly lower in DLB patients than AD patients in the scene pareidolia test (**S2 Table**), and the inter-rater agreement on the scene pareidolia test was excellent (0.99). These findings suggest that cognitive fluctuations in DLB patients and the manner of test administration, including instructions and feedback, may be the critical factors of inter-rater/test-retest variability in the scene pareidolia test.

The setting of cut-off scores for quantitative cognitive tests is helpful from a differential diagnosis perspective. The ROC analyses indicate that the optimal cut-off for the pareidolia score is 4/5 for the differentiation of DLB from AD and 2/3 for the differentiation of DLB and HC. However, these cut-off scores were calculated based on a small number of patients with limited forms of dementing disorders and with a narrow range of severity. Uncritical applications of these cut-off scores to individual patients may lead to an erroneous diagnosis and inappropriate treatment. Scores that are just below or above the cut-off scores should be interpreted with consideration of other clinical information. For example, if a multidomain non-amnestic mild cognitive impairment patient who exhibits prominent cognitive fluctuations scores 2 on the pareidolia score, a false negative may be considered. By contrast, a clinician may consider the possibility of a false positive when a severely amnesic dementia patient scores 6 without any other DLB-like clinical data. The validity of the cut-off scores proposed here, and the utility of the pareidolia test in a broader sense, should be examined in studies with a larger number of patients and in more diverse patient population including other types of dementias and psychiatric illnesses.

## Supporting Information

S1 Appendix Neuropsychological results of individual participants. (XLSX)

S1 Fig Performance on the pareidolia test in DLB patients with hallucinations and in those without hallucinations. (A) Illusory responses on the scene pareidolia test. (B) Illusory responses on the noise pareidolia test. (C) The pareidolia score. Significance is denoted by an asterisk (Mann-Whitney U test, p < 0.05).
(TIF)

S1 Table Results of the scene pareidolia test according to different scoring procedures. The number of images with illusory responses and total number of illusory responses were used in the current and previous studies, respectively.
(PDF)

S2 Table Numbers of patients who scored above or below the cut-off scores on the pareidolia score. (PDF)

S3 Table Intra-class correlation coefficients (ICCs) for the pareidolia test between patients with dementia with Lewy bodies and patients with Alzheimer's disease. (PDF)
