# Supplementary figures and images for: The Pareidolia Test: A Simple Neuropsychological Test Measuring Visual Hallucination-Like Illusions
Source: PLoS One. 2016 May 12;11(5):e0154713. doi: 10.1371/journal.pone.0154713 (PMC4865118; doi:10.1371/journal.pone.0154713)

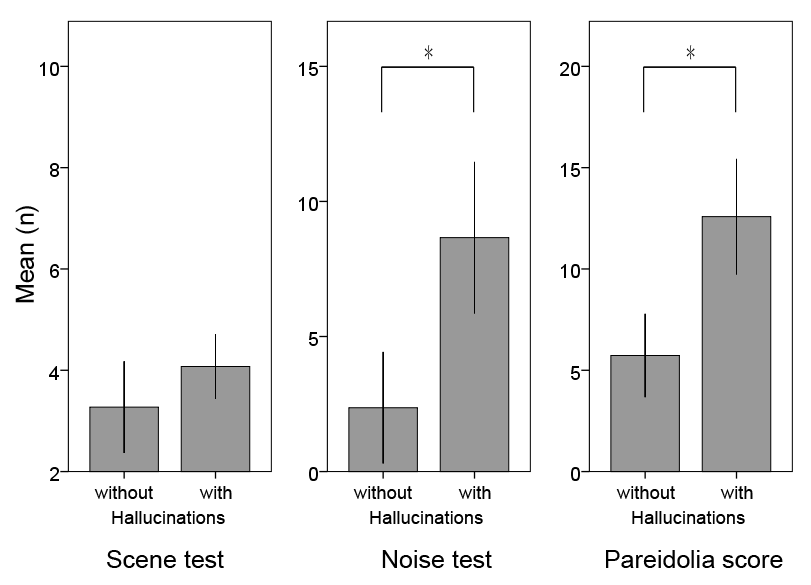

Supplement: S1 Fig — (A) Illusory responses on the scene pareidolia test. (B) Illusory responses on the noise pareidolia test. (C) The pareidolia score. Significance is denoted by an asterisk (Mann-Whitney U test, p < 0.05). (TIF) [file pone.0154713.s002.tif]
