# Supplementary material for: The Pareidolia Test: A Simple Neuropsychological Test Measuring Visual Hallucination-Like Illusions
Source: PLoS One. 2016 May 12;11(5):e0154713. doi: 10.1371/journal.pone.0154713 (PMC4865118; doi:10.1371/journal.pone.0154713)
Supplement: S1 Table — The number of images with illusory responses and total number of illusory responses were used in the current and previous studies, respectively. (PDF) [file pone.0154713.s003.pdf]

S1 Table. Results of the scene pareidolia test according to different scoring procedures. The number of images with illusory responses and total number of illusory responses were used in the current and previous studies, respectively.

	Current scoring procedure	Previous scoring procedure
The number of illusory responses (DLB/AD)	3.9±1.9/1.4±1.3*	6.2±4.5/2.1±2.2*
Test-retest/inter-rater reliability [a]	0.5	0.48
Correlation with the NPI hallucinations score (r_s) [b]	0.17	0.18
Differentiation between DLB and AD; Sensitivity/Specificity (Cut-off score) [c]	0.92/0.58 (1/2)	0.96/0.50 (1/2)

[a] Intra-class correlation coefficient; [b] Spearman's rank correlation; [c] Receiver operating curve characteristic analysis. Significance is denoted by an asterisk (Mann-Whitney U test, $p < 0.05$).
