# Supplementary material for: The Pareidolia Test: A Simple Neuropsychological Test Measuring Visual Hallucination-Like Illusions
Source: PLoS One. 2016 May 12;11(5):e0154713. doi: 10.1371/journal.pone.0154713 (PMC4865118; doi:10.1371/journal.pone.0154713)
Supplement: S2 Table — (PDF) [file pone.0154713.s004.pdf]

S2 Table. Numbers of patients who scored above or below the cut-off scores on the pareidolia score.

	DLB	AD
Pareidolia score (+)	44	3
Pareidolia score (-)	8	49
Total	52	52

The cut-off scores of the pareidolia score was 4/5.
