# Supplementary material for: The Pareidolia Test: A Simple Neuropsychological Test Measuring Visual Hallucination-Like Illusions
Source: PLoS One. 2016 May 12;11(5):e0154713. doi: 10.1371/journal.pone.0154713 (PMC4865118; doi:10.1371/journal.pone.0154713)
Supplement: S3 Table — (PDF) [file pone.0154713.s005.pdf]

S3 Table. Intra-class correlation coefficients (ICCs) for the pareidolia test between patients with dementia with Lewy bodies and patients with Alzheimer's disease.

	DLB (n=15)	AD (n=15)
Scene test	0.27	0.58
Noise test	0.79	0.89
Pareidolia score	0.78	0.84
